# Miliary Tuberculosis with Extrapulmonary Tissue Involvement

**DOI:** 10.1590/0037-8682-0101-2024

**Published:** 2024-05-27

**Authors:** Kemal Buğra Memiş, Bircan Beyza Korkmaz, Sonay Aydın

**Affiliations:** 1 Erzincan University, School of Medicine, Department of Radiology, Erzincan, Turkey.

**FIGURE 1:**
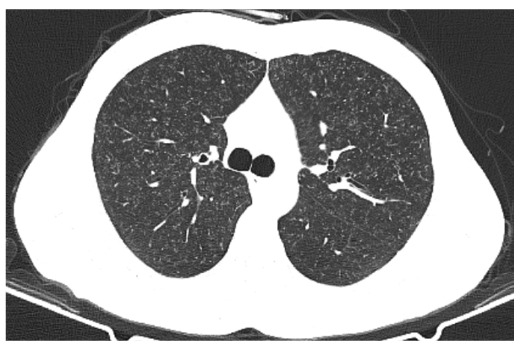
Axial noncontrast thoracic computed tomography image showing multiple micronodules randomly distributed in the bilateral lungs (miliary nodular pattern).

**FIGURE 2:**
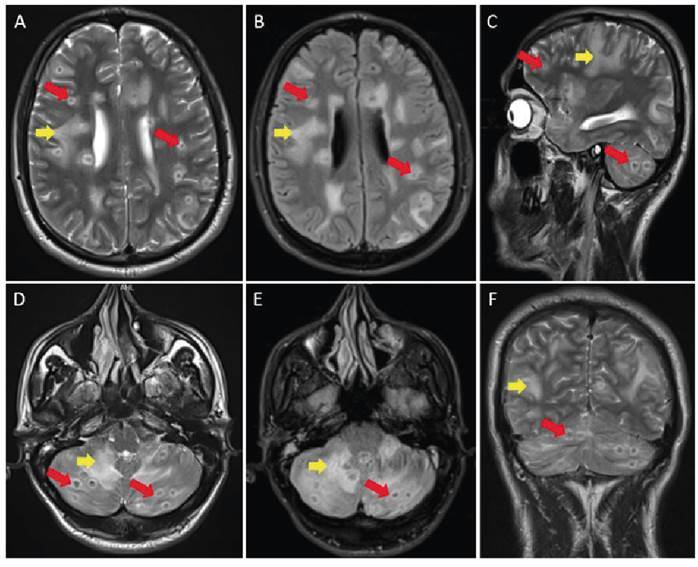
Axial T2-weighted (T2W) **(A, D)**, axial fluid attenuated inversion recovery (FLAIR) **(B, E)**, sagittal **(C),** and coronal **(F)** T2W brain magnetic resonance (MR) images showing lesion foci with a centrally hypointense and peripheral hyperintense signal intensity **(red arrows)**, which are numerous at the supra and infratentorial levels, most of which are subcortical, accompanied by peripheral vasogenic edema **(yellow arrows)** in some regions.

**FIGURE 3:**
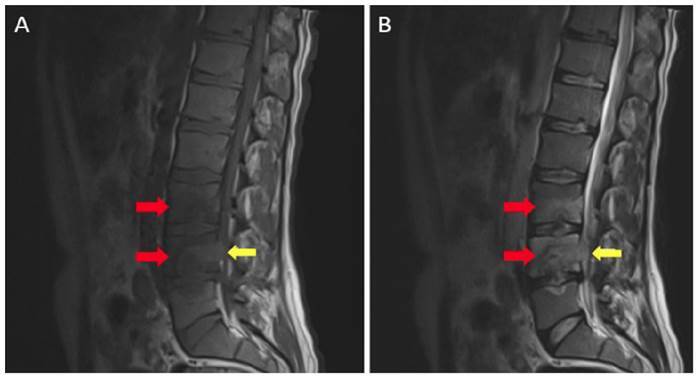
Noncontrast sagittal T1-wighted (T1W) **(A)** and T2-weighted (T2W) **(B)** lumbar magnetic resonance (MR) images show cortical irregularities accompanied by peripheral medullary bone marrow edema **(red arrows)** in the end plates adjacent to the L3-L4 and L4-L5 intervertebral discs. In addition, there are heterogeneous T2 signal increases in defined intervertebral discs. An abscess collection narrowing the spinal canal is also observed adjacent to the posterior corpus of the L4 vertebra **(yellow arrows)**.

A 37-year-old male patient visited the emergency department complaining of a cough. Thoracic computed tomography revealed the presence of widespread nodules, measuring 1-2 mm in diameter, with a miliary pattern in both lungs (Figure 1). After a diagnosis of miliary tuberculosis (TB) was confirmed through sputum culture, antituberculosis medication was initiated. During a noncontrast brain magnetic resonance imaging (MRI) scan conducted when the patient had a headache and tinnitus, several nodular lesions of different sizes were observed in the supratentorial and infratentorial white matter. These lesions, accompanied by edema, were identified as tuberculomas (Figure 2). Noncontrast lumbar MRI was performed because the patient complained of lower back pain, revealing the presence of spondylodiscitis at the L3-L5 level, which is indicative of Pott's disease and can lead to spinal canal compression (Figure 3).

TB is an infectious disease caused by *Mycobacterium tuberculosis*, which spreads through airborne particles. Although it primarily affects the respiratory system, it can also affect many other organs and systems of the body^1^. Brain tuberculoma is a granulomatous mass resulting from the hematogenous spread of TB. It is the most severe form of extrapulmonary TB^2^. Spinal TB has an insidious onset and commonly affects the thoracolumbar vertebrae and can lead to deformities and neurological deficits in complicated cases^3^. Imaging in patients diagnosed with TB can help detect possible extrapulmonary involvement early, preventing neurological impairment and other complications by prompt initiation of therapy.
